# Imaging biofilms using fluorescence *in situ* hybridization: seeing is believing

**DOI:** 10.3389/fcimb.2023.1195803

**Published:** 2023-05-22

**Authors:** Ana Barbosa, Sónia Miranda, Nuno F. Azevedo, Laura Cerqueira, Andreia S. Azevedo

**Affiliations:** ^1^ LEPABE - Laboratory for Process Engineering, Environment, Biotechnology and Energy, Faculty of Engineering, University of Porto, Porto, Portugal; ^2^ ALiCE - Associate Laboratory in Chemical Engineering, Faculty of Engineering, University of Porto, Porto, Portugal; ^3^ i3S-Instituto de Investigação e Inovação em Saúde, Universidade do Porto, Porto, Portugal; ^4^ IPATIMUP-Instituto de Patologia e Imunologia Molecular, Universidade do Porto, Porto, Portugal

**Keywords:** biofilms, fluorescence *in situ* hybridization (FISH), spatial organization of biofilms, multispecies biofilms, nucleic acid mimics

## Abstract

Biofilms are complex structures with an intricate relationship between the resident microorganisms, the extracellular matrix, and the surrounding environment. Interest in biofilms is growing exponentially given its ubiquity in so diverse fields such as healthcare, environmental and industry. Molecular techniques (e.g., next-generation sequencing, RNA-seq) have been used to study biofilm properties. However, these techniques disrupt the spatial structure of biofilms; therefore, they do not allow to observe the location/position of biofilm components (e.g., cells, genes, metabolites), which is particularly relevant to explore and study the interactions and functions of microorganisms. Fluorescence *in situ* hybridization (FISH) has been arguably the most widely used method for an *in situ* analysis of spatial distribution of biofilms. In this review, an overview on different FISH variants already applied on biofilm studies (e.g., CLASI-FISH, BONCAT-FISH, HiPR-FISH, seq-FISH) will be explored. In combination with confocal laser scanning microscopy, these variants emerged as a powerful approach to visualize, quantify and locate microorganisms, genes, and metabolites inside biofilms. Finally, we discuss new possible research directions for the development of robust and accurate FISH-based approaches that will allow to dig deeper into the biofilm structure and function.

## 1 Introduction

Microbial biofilm is considered one of the most widely distributed and successful way of life on Earth (Stoodley et al., 2002; Hall-Stoodley et al., 2004), being the predominating microbial lifestyle in most natural environments (Donlan and Costerton, 2002; Yan and Bassler, 2019). Biofilm is defined as sessile microbial consortia firmly attached to a surface with a three-dimensional structure, where multicellular microbial cells are embedded in a matrix composed of extracellular polymeric substances (EPS) (Flemming and Wingender, 2010; Azevedo et al., 2021). The EPS are mainly composed of polysaccharides, proteins, lipids, and extracellular nucleic acids (eRNA and eDNA) and play an important role in the biofilms’ structure and function, being an essential key for their emergent properties (Flemming et al., 2016; Di Martino, 2018).

The original 5-step model to describe this microbial consortia begins with a biofilm formation that involves highly complex and dynamic processes, where hydrophobic interactions, steric interactions, proteins adhesion, electrostatic interactions, and Van der Waal forces are mostly responsible for the adhesion of bacteria on surfaces (Khatoon et al., 2018). After that, some bacterial cells become irreversibly attached to the surface, followed by cell growth with extracellular matrix formation, development, and maturation of the biofilm three-dimensional (3D) architecture. Finally, some of the bacteria may be released into the liquid medium and colonize and form a new biofilm in a different place (Kostakioti et al., 2013; Tolker-Nielsen, 2015). However, this model fails to capture the multiple biofilm structures and phenotypes that can be formed with different bacteria and in different microenvironments. Saur et al., suggested a new overall model for biofilm formation based in three major events: aggregation, growth, and disaggregation, in order to introduce a common platform to improve the understanding of behavior of microorganisms in industrial systems, environmental habitats and medical settings (Sauer et al., 2022).

The biofilm state confers numerous ecological and physiological advantages to microorganisms, such as, exchange of metabolites, horizontal gene transfer, protection under stress conditions (e.g., nutrient deprivation, extreme temperature and pH), protection and resistance to antimicrobial agents (e.g., antibiotics, disinfectants, antiseptics), host immune response (e.g., antibodies, phagocytes) and shear forces (Stewart and Franklin, 2008; Burmølle et al., 2014; Srinivasan et al., 2021). However, depending on their location and the species involved, biofilms can be both beneficial or detrimental to human society (Hibbing et al., 2010). An example of a helpful biofilm application can be found in the wastewater treatment (Yamashita and Yamamoto-Ikemoto, 2014; Naidoo and Olaniran, 2013); on the other hand, biofilm-associated infections on animals and humans have a high impact on human livelihoods and economy (Wu et al., 2015; Subhadra et al., 2018).

In nature, biofilms typically involve a mixture of several microorganisms (Elias and Banin, 2012) that interact with each other and organize themselves into 3D structured communities (Roder et al., 2020). In fact, the species structural organization within biofilms is influenced by both local interactions between physiologically distinct species and larger-scale environmental factors (e.g., nutrient availability, ionic strength, pH, and temperature) (Booth and Rice, 2020; Monticolo et al., 2020). The characterization of these communities, not only at phylogenetic level, but also on their spatial and temporal interactions, are of the utmost importance, as it can influence the way to deal with them (Stacy et al., 2016; Azeredo et al., 2017). The rapid technological advancements in biofilm studies have improved our understanding of microbial communities structure, function, and response to environmental factors (Seneviratne et al., 2020). **Table 1** describes the most used technologies in biofilm research, including the “omics” technologies. Briefly, metagenomics and genomics studies using high-throughput DNA sequencing technologies (e.g., next-generation sequencing) have provided insight into the genetic coding potential of biofilm organisms and into biofilm community structures (Hasan et al., 2014; Rehman et al., 2020). Transcriptomics approaches, including RNAseq (Miller et al., 2018; Partoazar et al., 2019), microarrays (Folsom et al., 2010; Ebersole et al., 2019), and quantitative reverse transcription polymerase chain reaction (RT-qPCR) (Magalhaes et al., 2019; Partoazar et al., 2019) have advanced our understanding of global and localized gene expression processes that occur within biofilms. However, the application of these methodologies implies the disruption of the biofilm, thus losing the possibility of observing *in situ* the different species and components. In fact, one of the most important advances in the study of the structure and dynamics of biofilms has been the ability to spatially locate and detect different species or particular components (e.g., genes) within biofilm, without disrupting it (Costa et al., 2017; Ramírez-Puebla et al., 2022). Within this context, *in situ* fluorescence imaging approaches offer promising opportunities to visualize the structure of biofilms and clarify the function of microorganisms.

**Table 1:** Molecular techniques: a brief definition, application, and some biofilms studies.

Technique	Brief definition	Application	Reference
Next-generation sequencing (NGS)	The Next Generation sequencing (NGS) approach can provide billions of nucleotides of sequence for an individual sample with higher sensitivity, faster turnaround time, and lower cost (Reuter et al., 2015; van Dijk et al., 2018). The high-throughput sequencing technologies, such as Illumina, Roche 454, SOLiD, and Pacific Biosciences [PacBio] have dramatically increased sequencing capabilities (Franklin et al., 2015).	The total DNA or collective genome (metagenome) is isolated from the environment and then sequencing is applied to provide a comprehensive view of the genetic diversity, species composition, evolution, and interactions with the environment of natural microbial communities (Handelsman, 2004).	(Hasan et al., 2014; Eriksson et al., 2017; Pereira et al., 2018; Rehman et al., 2020)
RT-qPCR	The quantitative polymerase chain reaction (qPCR) it’s based on the detection of fluorescence during the PCR reaction and allows analysis of the transcriptome (Lowe et al., 2017). The qPCR method allows real-time monitoring of gene expression is rapid and relatively low cost, it can target only known sequences and only a few targeted genes can be investigated (Franklin et al., 2015).	The RT-qPCR method allows real-time monitoring of gene expression and can be used to detect gene expression changes in the biofilm state or after exposure to compounds (Partoazar et al., 2019). This technology has also been used for the quantification of biofilm viable organisms (Azeredo et al., 2017) and can be used to confirm or validate microarray/RNA-Seq results (Shemesh et al., 2007).	(Zemanick et al., 2010; Magalhaes et al., 2019; Partoazar et al., 2019)
Microarrays	Microarray analysis is based on fixed thousands of probes to o a surface and samples are labeled with fluorescent dyes for detection after hybridization (Lowe et al., 2017; Kinaret et al., 2020). The fluorescence intensity at each probe location on the array is measured and indicates the transcript abundance for that probe sequence (Lowe et al., 2017).	Microarrays can analyze the expression levels of several genes, providing information about a specific response at a given time (Seneviratne et al., 2020). Also, can be used to profile differentially expressed genes and identify markers capable of distinguishing cells (Franklin et al., 2015; Dai and Shen, 2022).	(Resch et al., 2005; Folsom et al., 2010; Ebersole et al., 2019)
RNA-seq	High-throughput RNA sequencing (RNA-Seq) is a standard technique for transcript discovery and differential gene expression analysis in life science laboratories (Stark et al., 2019). This technology provides information that may not be available with the microarray approach, such as the presence of small noncoding RNAs expressed from intergenic regions and information on the sites of promoter sequences and operon structures (Franklin et al., 2015).	The RNA-Seq allows the detection and quantification of both known and novel transcripts making it possible to analyze the entire transcriptome of biofilms (Lowe et al., 2017). In this sense, this approach provided unique insights into biofilm biochemical properties, environmental and genetic factors that influence biofilm formation (Peterson et al., 2014; Seneviratne et al., 2020)	(Miller et al., 2018; Partoazar et al., 2019; Wu et al., 2019; Peyrusson et al., 2020)

There is an increasing list of fluorescence imaging techniques and fluorescent dyes that offer the possibility to detect and locate the different biofilm components. For the staining of the matrix polysaccharides, there are some techniques that can be used depending on the polysaccharides to be identified and located (Schlafer and Meyer, 2017). To identify di- or trisaccharides that are present both in the biofilm matrix and on the cell surface, as glycoconjugates, (in the teichoic acids of Gram-positive bacteria and in the lipopolysaccharides of Gram-negative bacteria), fluorescently labelled lectins are used (Cruz et al., 2021). For β-1,3 and β-1,4 glucans (polysaccharides only found in cellulose and chitin) identification, it is used the calcofluor white staining (Sime-Ngando et al., 2022). There are still other techniques for polysaccharides identification, as the use of a specific modified green fluorescent protein (GFP) (Nguyen et al., 2014). Another important biofilm matrix component is the eDNA, that can be detected by several cell-impermeant DNA-binding fluorescent stains as propidium iodide (PI), 1,3-dichloro-7-hydroxy-9,9-dimethyl-2(9H)-acridinone (DDAO), TOTO^®^-1, TO-PRO^®^ 3, PicoGreen^®^ and SYTOX^®^ stains (Dutta et al., 2021). Still, to study proteins, another relevant compound of the biofilm matrix, fluorescently labeled antibodies (primary or secondary) can be used. It may also be studied using a FilmTracer™ SyPro^®^ stains (Frank and Patel, 2007; Berk et al., 2012). Lipids, in it turns, can be identified by Nile red, hydrophobic BODIPY^®^ dyes and carbocyanine DiD (Dutta et al., 2021; Schlafer and Meyer, 2017). In addition, the LIVE/DEAD assay (mixture of SYTO9 and propidium iodide (PI)), has proven useful for the *in situ* viability estimation, based on membrane integrity; combining LIVE/DEAD assay with confocal laser scanning microscopy (CLSM) analysis, the location of live bacteria with intact membranes (green) and dead bacteria with compromised membranes (red) can be visualized (Nistico et al., 2014). However, Rosenberg et al., have suggested that the presence of extracellular nucleic acids (stained red by PI) in the biofilm matrix might overestimate the dead PI-staining biofilm cells (Rosenberg et al., 2019). Regarding the identification and location of microorganisms within biofilms, two well-known fluorescent methods were developed: the fluorescent protein (FP) labeling and the fluorescence *in situ* hybridization (FISH). In FP method, microorganisms are genetically modified to produce fluorescent proteins. It is a non invasive imaging technique, allowing the evaluation of biofilms’ biological activity in real-time, without any previous treatment. However, it is not amenable to natural environments because genetic modifications of the microorganisms are needed (Costa et al., 2017). Hence, FISH has emerged, as a powerful tool, for the detection (identification and quantification) of microorganisms, analysis of the genome, the transcriptome and the spatial distribution of biofilms in their natural environment (Moter and Gobel, 2000; Huber et al., 2018).

## 2 FISH to spatially locate microorganisms in biofilms

The emergence of FISH became crucial to better understand inter-species interactions, allowing to identify and observe the location of different microorganisms directly on biofilm, without disturbing their 3D structure (Brileya et al., 2014). In this technique, a fluorescently labeled sequence-specific complementary probe (typically a DNA probe) will hybridize with their nucleic acid target (e.g., DNA, mRNA, rRNA) inside cells or tissues. Typically, sequences of 16S/23S and 18S/28S rRNA are the preferential target for members of the Bacteria/Archaea and Eukarya domains, respectively, as these are universal and highly abundant. They are also composed of highly conserved regions as well as variable ones, which allows the design of probes with different cell specificity (Stewart and Franklin, 2008; Patwardhan et al., 2014; Nácher-Vázquez et al., 2022).

**Figure 1:**
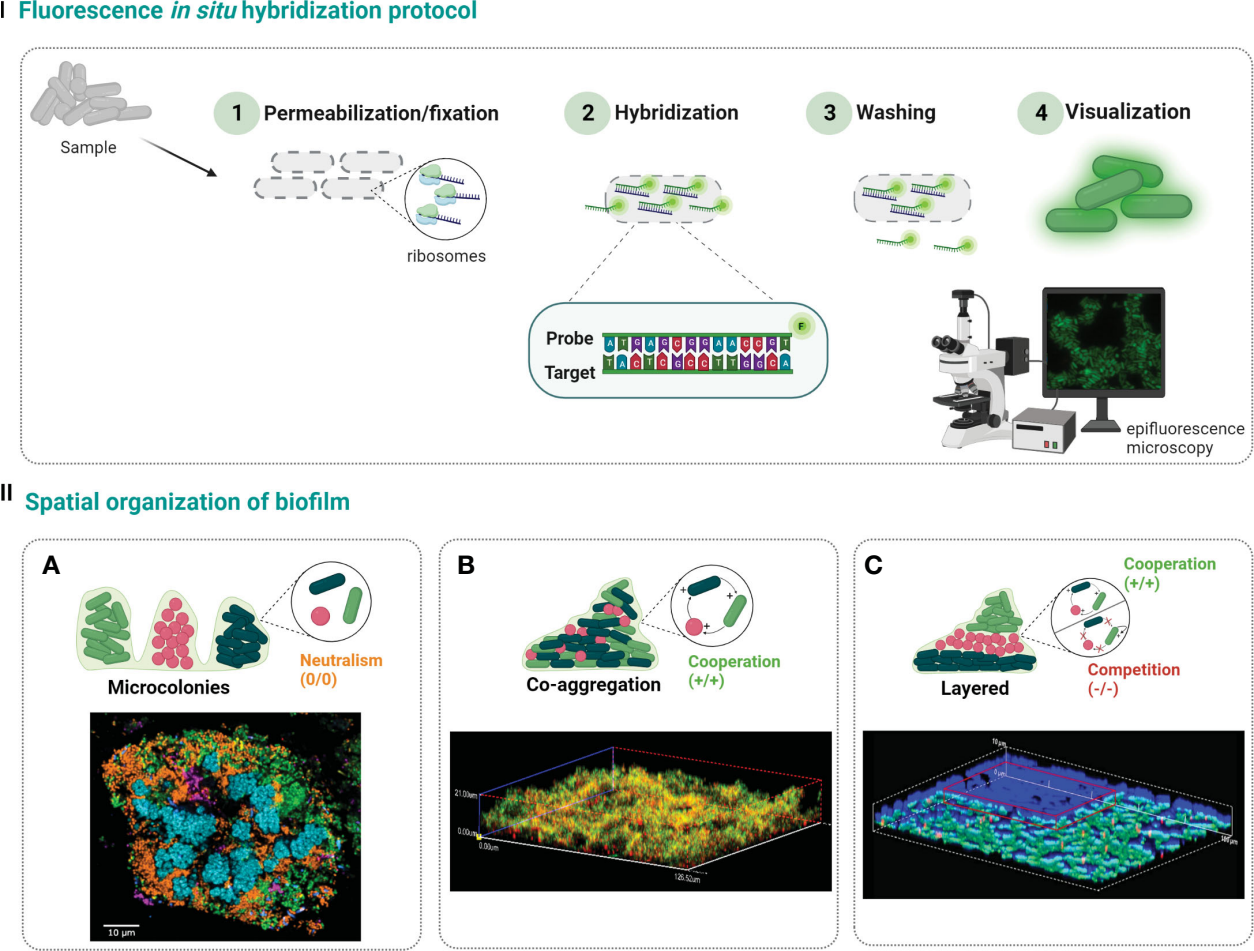
Steps of traditional FISH protocol (I). First, the biofilm sample is fixed to stabilize the cells and permeabilize the cell membrane (1). Then, labelled probes are added and allowed to hybridize with the rRNA target (2), and the excess probe is washed away (3). Finally, the sample is analyzed under epifluorescence microcopy or confocal laser scanning microscopy to determine the spatial distribution of biofilm populations (4). Species in polymicrobial biofilms can be organize in three different ways (II): **(A)** separate microcolonies (confocal microscopy image reprinted with permission from (Mark Welch et al., 2016)); **(B)** co-aggregation (confocal microscopy image reprinted with permission from (Azevedo et al., 2016)); **(C)** arranged in layers (confocal microscopy image reprinted with permission from (Almeida et al., 2011)). Created with BioRender.com.

Probe design is indeed one crucial step that can influence the FISH performance. For that, it is important to consider specificity and sensitivity when selecting a FISH-probe; a high specificity of the probe means that it might correctly discriminate the target from nontarget species (Rocha et al., 2019). On the other hand, a high sensibility refers to the ability of the probe to detects all strains of the taxonomic group for which it was designed (Rocha et al., 2019). Besides the theoretical value of specificity and sensitivity, there are other important criteria that must be considered in the probe design, such as, length of the probe, GC percentage, melting temperature, and number of mismatches with close sequences (Teixeira et al., 2021). Briefly, the probe length and GC content (due to the effect of the GC triple hydrogen bonds) influences the melting temperature (temperature at which 50% of the double-stranded of nucleic acid strands is changed to single-stranded) (Almeida and Azevedo, 2021; Teixeira et al., 2021). The effect of GC content is more pronounced when the probes are shorter, and it is recommended should be between 40% and 60% (Aquino de Muro, 2008; Almeida and Azevedo, 2021). In addition, the probe length also influences the diffusion of the probe through cellular envelope (a shorter the probe provides a better the diffusion) and its discrimination power (a shorter the probe provides higher the discrimination). However, the discrimination power may not be translated in a higher specificity; in fact, if the probe is too small, it also increases the odds of the target sequence being found in other organisms(Almeida and Azevedo, 2021). As so, the length of the probe should be between 12 and 20 bp, depending on the nature of the probe; for instance, nucleic acid mimics probes usually need shorter probes (12-15 bp) (Cerqueira et al., 2008; Teixeira et al., 2021). Finally, the FISH performance may also be affected by the number of mismatches found in sequences of the nontarget organisms. The presence of mismatches delays the hybridization rate and, therefore, the designed probe should present none mismatches for the target sequences and, as much as possible mismatches for the nontarget sequences (Almeida and Azevedo, 2021).The FISH protocol involves the following four steps (**Figure 1**-I): 1) cells fixation and permeabilization, 2) hybridization of the probe with the target, 3) washing the residual probe, and 4) visualization of the fluorescence emitted by hybridized cells. In the first step, chemical fixatives commonly used in bacterial and human cells are used, to inactivate enzymes and stabilize nucleic acids’ structures. Next, for the probes to access and hybridize with the target, some parameters, as temperature, pH, ionic strength and formamide concentrations, must be well-defined. The next step is the sample washing to remove all loosely bound or unbound labelled probes, conferring a higher detection specificity. Lastly, but still of huge importance, the result of hybridization is visualized by epifluorescence microscopy or CLSM (Cerqueira et al., 2008; Almeida and Azevedo, 2021; Nácher-Vázquez et al., 2022).

There are three main patterns of microbial spatial organization on biofilms that can be observed when FISH is applied (**Figure 1**-II): a) single cell microcolonies, where each species is in separated microcolonies, showing a non-commensal or neutral interactions (Nielsen et al., 2000); b) co-aggregation, where the different species are all mixed and can be found together throughout the biofilm with a cooperative behavior (Azevedo et al., 2016; Allkja et al., 2022); and c) the layered organization, where one species can be found in the lower layer of biofilm and the other in the upper layer, which might be related with both cooperative or competitive relations (Habimana et al., 2010; Almeida et al., 2011).

In the following sections, we describe different FISH variants already applied for the *in situ* visualization of biofilms; their applicability, advantages and limitations on biofilm research are also discussed. The first group focuses on FISH variants that emerged to improve the FISH robustness for the spatial organization analysis of biofilms (e.g., in terms of fluorescence intensity signal and probe diffusion thought the biofilm matrix); the second group discusses multiplexed FISH approaches for the visual characterization of multispecies biofilms; and in the last group, FISH variants for the study of metabolic activity of biofilm cells are described. Lastly, recent and innovative FISH approaches are also presented and discussed.

### 2.1 FISH-based techniques applied to biofilms

Despite of obvious advantage of FISH, when combined with CLSM, in terms of spatially discrimination and detection of microorganisms within biofilms, the high background fluorescence due to non-specific adherence of long-fragment DNA probes and the low signal intensity due to the low ribosomal RNA (rRNA) content or insufficient accessibility of the target molecule, can hinder classical FISH methodologies (Azevedo et al., 2022). In fact, in biofilms studies, the cells are not displayed in one layer but embedded in a 3D extracellular matrix. The bottom cells are usually less active (due to their inability to receive all the nutrients and oxygen that they need to survive) (Røder et al., 2021), which implies less copies of rRNA and a lower signal intensity that might not be easily observable by epifluorescence microscopy. In addition, the matrix that involves the biofilm cells behaves as a barrier to outside elements (such as, antibiotics and toxins), which will hinder the diffusion of the probes (Dufour et al., 2010). Hence, catalyzed reporter deposition (CARD)-FISH and nucleic acid mimics-FISH (NAM-FISH) have emerged to improve the performance of FISH for the *in situ* visualization of biofilms. On the other hand, another concern on the study of biofilm dynamics is that the spatial organization and composition of biofilm evolves over time. As such, the fluorescence *in vivo* hybridization (FIVH) has tackled this challenge by providing *in vivo* information on the changes occurring in the biofilm (Fontenete et al., 2016).

#### 2.1.1 CARD-FISH

**Figure 2:**
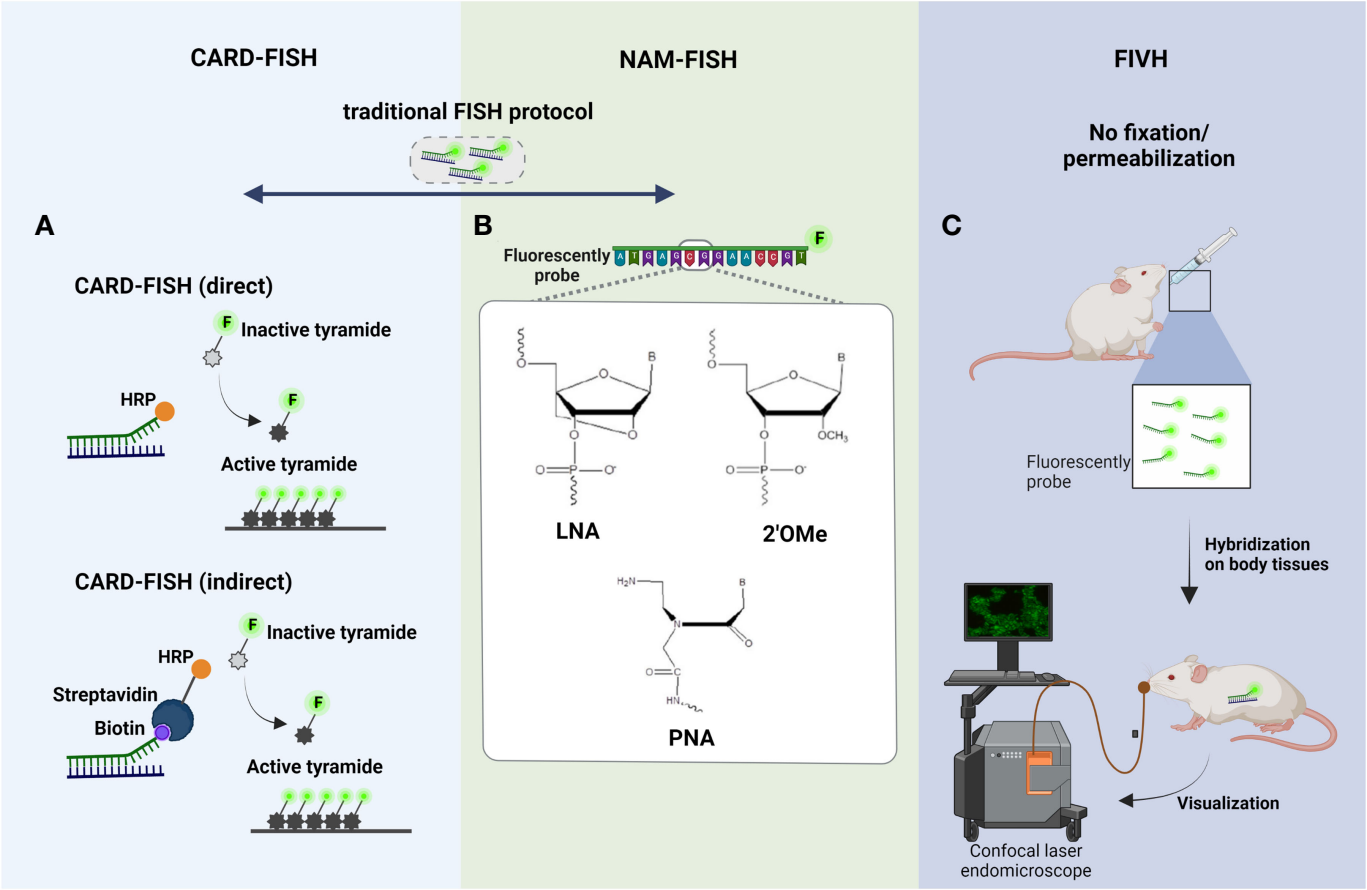
Principle steps of **(A)** CARD-FISH, **(B)** NAM-FISH and **(C)** FIVH. The four steps of traditional FISH are the basic standpoints of these modified FISH variants, being highlighted in the figure the main improvements made on the hybridization step. **(A)** In direct CARD-FISH, horseradish peroxidase (HRP) conjugated probes hybridized with the complementary rRNA sequence, and signal amplification is conducted with fluorescein labeled tyramides; on the other hand, in the indirect CARD-FISH, biotinylated probes and HRP-labeled streptavidin are used; **(B)** In NAM-FISH panel is showed the chemical structure of the most used NAMs (LNA, PNA and 2’OMe); **(C)** In FIVH protocol, the fixation and permeabilization step is eliminated and the NAM probe is given by oral gavage; then, the hybridization occurs inside of mouse body, at normotemperature (37°C), and the visualization of fluorescent cells can be performed directly using a confocal laser endomicroscope. Created with BioRender.com.

As already referred, the metabolic activity of species can vary depending on their location within biofilm, resulting from nutrient concentration gradients; this means that cellular rRNA content of species can also significantly differ (Melaugh et al., 2016). As such, discrepant fluorescence FISH signals between cells in different layers is expected. In addition, minimum amounts of rRNA might be more pronounced in environmental microorganisms (e.g., from lakes, rivers, soil, rocks covering, sludge, marine water, sediments), which typically grow under specific nutrient-limiting conditions (Hoehler and Jørgensen, 2013; Matturro et al., 2021). Therefore, CARD-FISH, also known as tyramide signal amplification (TSA)-FISH, was developed to amplify the FISH signal, being one of the most important molecular tools for the detection of environmental microorganisms (e.g., in soil, rocks, sludge, marine water) (Kubota, 2013). There were developed two different variants of CARD-FISH: a direct method using probes directly linked with horseradish peroxidase (HRP) and an indirect method using biotinylated probes and HRP-labeled streptavidin (Kubota, 2013) (**Figure 2A**). CARD-FISH has been shown to increase the sensitivity 26- to 41- times than standard FISH (Hoshino et al., 2008). For instance, Ferrari et al., showed that CARD-FISH improved the detection of soil bacteria comparing with conventional FISH (Ferrari et al., 2006). Hence, the application of CARD-FISH on analysis of environmental biofilms showed as a promising tool. Fernandez et al., investigated the bacterial diversity in biofilms from chemolithotrophic denitrifying bioreactors. In this study, CARD-FISH results showed that bacterial diversity in the chemolithotrophic denitrifying bioreactor changed significantly during the initial period of operation with the dominance of *Actinobacteria* and *Firmicutes*; and, after 6 months of operation the bacterial diversity become unaltered with the dominance of α, β and γ-*Proteobacteria* (Fernandez et al., 2008). Later, Lupini et al., assessed the spatial distribution of α, β and γ-*Proteobacteria* in a riverine biofilm; the results showed that the bacteria are co-aggregated with the dominance of α-*Proteobacteria* (Lupini et al., 2011). In 2018, Gregorio et al., have used CARD-FISH to analyze the biofilm dynamics from an open full-scale cooling tower. Biofilms collected in the summer showed that *Diadesmis* sp., filamentous cyanobacteria and green algae are co-aggregated within the diatoms (Di Gregorio et al., 2018).

This technique could also be a promising tool on biofilm research in different areas, including the industrial (such as in food processing) and clinical areas. Nevertheless, the permeabilization in CARD-FISH can be the limiting step, since cell penetration of probes labeled with HRP (5-6 nm and 40 kDa of molecular weight, approximately) is less effective when compared with smaller molecules as traditional probes labeled with fluorochromes (500 – 1000 Da of molecular weight, approximately) (Hoshino et al., 2008). Therefore, optimization of the permeabilization step in biofilm samples is critical in this method. The size of HRP also represents a concern in biofilms since the biofilm matrix might hinder its diffusion. In addition, as the CARD reaction is catalyzed by a peroxidase, an extra step before hybridization should be added to inactivate endogenous peroxidases expressed during biofilm formation (Kubota, 2013).

#### 2.1.2 NAM-FISH

While some improvements on conventional FISH have been made individually with CARD-FISH technique, in terms of detection of microorganisms with low rRNA content, there was still diffusion limitations of probes through the biofilm matrix. In fact, biofilm matrix is composed of a complex array of EPS (Flemming et al., 2016) negatively charged which might hinder the diffusion of DNA probes (also negatively charged). In addition, the use of DNA probes on FISH technique can also present other shortcomings that might compromise the fluorescence signal, specificity and sensitivity of the probes, including low affinity and target site accessibility and poor cell permeability (Yilmaz and Noguera, 2004; Yilmaz et al., 2006). To overcome these problems, chemically modified DNA and RNA probes, commonly known as nucleic acid mimics (NAMs), were developed, still obeying to the Watson-Crick base-pairing rules (Cerqueira et al., 2008). The chemical modifications of NAMs might be at nucleobase, sugar ring or phosphodiester backbone level (Karkare and Bhatnagar, 2006). Nowadays, there are available different NAM probes, but the most used are the Peptide Nucleic Acid (PNA), Locked Nucleic Acid (LNA) and 2’-O-Methyl-RNA (2’OMe) (**Figure 2B**). PNA is an oligonucleotide that has a neutral peptide backbone instead of the DNA/RNA negatively charged sugar–phosphate backbone. PNA is stable molecule as it is resistant to hydrolytic (enzymatic) cleavage. The stability and specificity on the hybridization process, when compared to DNA/RNA molecules, is also higher, as the neutrally charged backbone lowers the repulsive eletrostatic interactions between the PNA/DNA or PNA/RNA duplexes (Saarbach et al., 2019). LNA is a synthetic RNA which offers several advantages such as greater affinity toward DNA and RNA targets, higher biostability (resistance to nuclease degradation), better signal-to-noise ratio and better sensitivity and specificity (Petersen and Wengel, 2003; Silahtaroglu et al., 2004; Thomsen et al., 2005; Veedu and Wengel, 2009). 2’OMe is another RNA mimic which displays a high nuclease resistance and a greater stability and specificity for RNA targets than the LNA or DNA probes (Kierzek et al., 2005). The choice of the chemical nature of the probe (DNA *vs* NAM) is done according the application and aim of the study, resulting in a great impact on the robustness of FISH protocol. In fact, PNA-FISH combined with CLSM has been extensivley applied in biofilm studies (some examples, (Malic et al., 2009; Almeida et al., 2011; Cerqueira et al., 2013; Lopes et al., 2017; Azevedo et al., 2020; Sousa et al., 2021), since a better diffusion of PNA through the biofilm matrix can occur due the lack of charge repulsion between the neutral PNA strand and the components of matrix (Stender et al., 2002). In these studies, was proven that with PNA-FISH is possible to observe the different types of spatial organization schematized in **Figure 1**-II. For instance, Almeida et al., employed PNA-FISH to quantify and visualize multispecies biofilms formed by *Salmonella enterica*, *Listeria monocytogenes* and *Escherichia coli* in different support materials (e.g., glass, polyvinyl chloride, copper, stainless steel). The CLSM images revealed that the 3 species are organized in two well-defined layers, in which *E. coli* is on the upper layer exclusively (showing an antagonistic behavior) and *S. enterica* and *L. monocytogenes* are mixed on the bottom of biofilm (**Figure 1**-II). In 2020, Azevedo et al. applied a PNA-FISH protocol in a dual-species biofilm formed by two different *E. coli* strains, in conditions mimicking the urinary infections, showing a coaggregation structure/cooperative strategy (Azevedo et al., 2020). In another study carried out by Sousa et al., the potential of PNA-FISH for the *in situ* discrimination of bacterial vaginosis-associated pathogens (*Fannyhessea vaginae* and *Gardenerella vaginalis*) was confirmed (Sousa et al., 2021).

Concernig the LNA and 2’OMe probes, there are just a few studies regarding their application to biofilms (Azevedo et al., 2015; Azevedo et al., 2016; Allkja et al., 2022), and even these are limited for the *in situ* detection and localization of biofilm populations to assess the spatial organization. Despite of negative charge of LNA and 2’OMe probes, all these studies have shown a complete staining of the biofilm sample and a strong fluorescence signal, even in thicker biofilms (Azevedo et al., 2015); it might be possible that the other characteristics (e.g., higher water solubility, more efficient hybridization) of LNA and 2’OMe (Braasch and Corey, 2001; Elayadi et al., 2002; Robertson and Thach, 2009) are also determinant, assuring an efficient spatial characterization of biofilm populations. For example, Azevedo et al., using LNA/2’OMe-FISH combined with CLSM, observed that *E. coli* and atypical bacteria (*Delftia tsuruhatensis* and *Achromobacter xylosoxidans*) are well mixed and aggregated to each other, resulting in a cooperative bahaviour after antibiotic treatment (**Figure 1**-II) (Azevedo et al., 2016). Still, a recent study from Allkja et al., applying LNA/2’OMe-FISH, observed that *Enterococcus faecalis*, *E. coli*, *Candida albicans*, and *Proteus mirabilis* (species commonly associated with catheter-associated urinary tract infections), formed a co‐aggregated structures typical of a cooperative relationship (Allkja et al., 2022). Yet, another application of LNA-FISH, was demonstrated by Vilas Boas et al., for the *in situ* analysis of bacteriophage-bacteria interactions in biofilm structures; these authors have easly detected and discriminated phage-infected and noninfected cells in biofilms formed by *Pseudomonas aeruginosa* and *Acinetobacter baumannii*, applying LNA probes to target a conserved gene highly expressed during multiplication of bacteriophage inside the bacterial cells (Vilas Boas et al., 2016).

However, an important drawback of NAM-FISH is the low number of distinguishable targets in a single experiment. In fact, despite the conceptual possibility design of specific probes for almost any microorganism, the number of fluorochromes that can be simultaneously differentiated is restricted to 3/4, due to the use of band- or long-pass filters in epifluorescence microscope, which does not allow to separate fluorochromes with highly overlapping excitation and emission spectra (Valm et al., 2012; Zimmermann et al., 2014). In addition, the NAM-FISH protocol does not operate in real-time since it involves a fixation/permeabilization step, which compromise the cells membrane and the cells are no longer viable (Amann and Fuchs, 2008). This is an important issue when the assessment of composition and species localization over time inside biofilm, is intended.

#### 2.1.3 FIVH

The biofilm development is a continuous and dynamic interplay between microorganisms themselves and/or between microorganisms and host. Hence, *in vivo* biofilm analysis has become essential to understand the changes occurring during biofilm development and maturation and also to monitor the response to different stimuli, such as, antimicrobial agents, modified surfaces, shear stress, pH and temperature changes, supply of nutrients, immune molecules produced by the host (Guzmán-Soto et al., 2021; Funari and Shen, 2022). For that, conventional FISH was adapted for *in vivo* purposes; there was a need to eliminate the fixation and permeabilization step since the chemical compounds (ethanol, methanol, paraformaldehyde) used are toxic to cells (Batani et al., 2019). Additionality, the probes must hybridize at room temperature or at human body temperature (e.g., normotemperature, 37°C) under non-toxic conditions (e.g., without formamide in the hybridization solution) (Fontenete et al., 2013) (**Figure 2C**). FIVH emerged to efficiently detect microorganisms inside the human body or the body of other higher-order animals (Fontenete et al., 2013). Fontenete et al., have optimized a non-toxic FISH protocol for the *in vivo* detection and location of *Helicobacter pylori* directly on a biofilm naturally found in the stomach of mice. The LNA/2’OMe probe used in FIVH protocol has not only to work efficiently at 37°C without toxic chemical compounds, but also in the presence of gastric juice and low pH, as observed in stomach mucosa (Fontenete et al., 2016). In fact, NAMs showed to be promising molecules, especially for *in vivo* applications, in which the probes must work at a specific temperature (e.g. human body temperature) (Fontenete et al., 2013; Fontenete et al., 2016). Recently, Moreira et al., have showed that the use of nanoparticles, as liposomes, might help in the delivery of NAM probes into cells for the visualization of spatial distribution of microorganisms *in vivo*, without time-consuming fixation and permeabilization steps (Moreira et al., 2022). In future, FIVH will offer the possibility of a spatial and temporal distribution analysis of more complex biofilms, including biofilms found in humans, animals, plants, soils and aquatic environments, contributing to more a reliable and realistic analysis. Nonetheless, FIVH was never employed to detect multiple targets within the human body due to the lack of suitable systems that were able to detect fluorescence signals in real-time. For instance, medical devices with built-in advanced imaging systems (e.g., confocal laser endomicroscopy) allow an analysis of stomach and colon (Polglase et al., 2005; Miller et al., 2011). However, the confocal laser endomicroscopy available in the market have an excitation wavelength of 488 nm (Polglase et al., 2005), thus, it does not allow to observe multiple targets simultaneously. On the other hand, maestro *in-vivo* imaging system is a multispectral imaging fluorescence-based methodology, that can be used for the *in vivo* imaging of multiple fluorochromes in small animals (Mansfield et al., 2005; Levenson et al., 2008). Nonetheless, it has already been used to study infections, there are many drawbacks that still need to be circumvented, such as finding ways to improve their resolution - the resolution of these tools is high (25 microns/pixel), but it is not enough to identify single microorganisms (Levenson et al., 2008).

### 2.2 Multiplex FISH-based techniques applied on multispecies biofilms studies

In almost all natural settings, biofilms are multispecies which highlights the importance of studying multispecies biofilm architecture and its influence on dynamics of microorganisms. Although, the study of single-species biofilms is more comprehensive, bacterial social interactions in multispecies biofilms have been gathering scientific interest and therefore, multispecies’ studies in biofilms are increasing. As so, multiplexed FISH methodologies, including double-labeling-of-oligonucleotide (DOPE)-FISH (Schimak et al., 2016) and combinatorial labeling and spectral imaging (CLASI)-FISH, emerged to increase the number of different species/targets detected in a unique biofilm sample (Valm et al., 2016).

#### 2.2.1 DOPE-FISH

**Figure 3:**
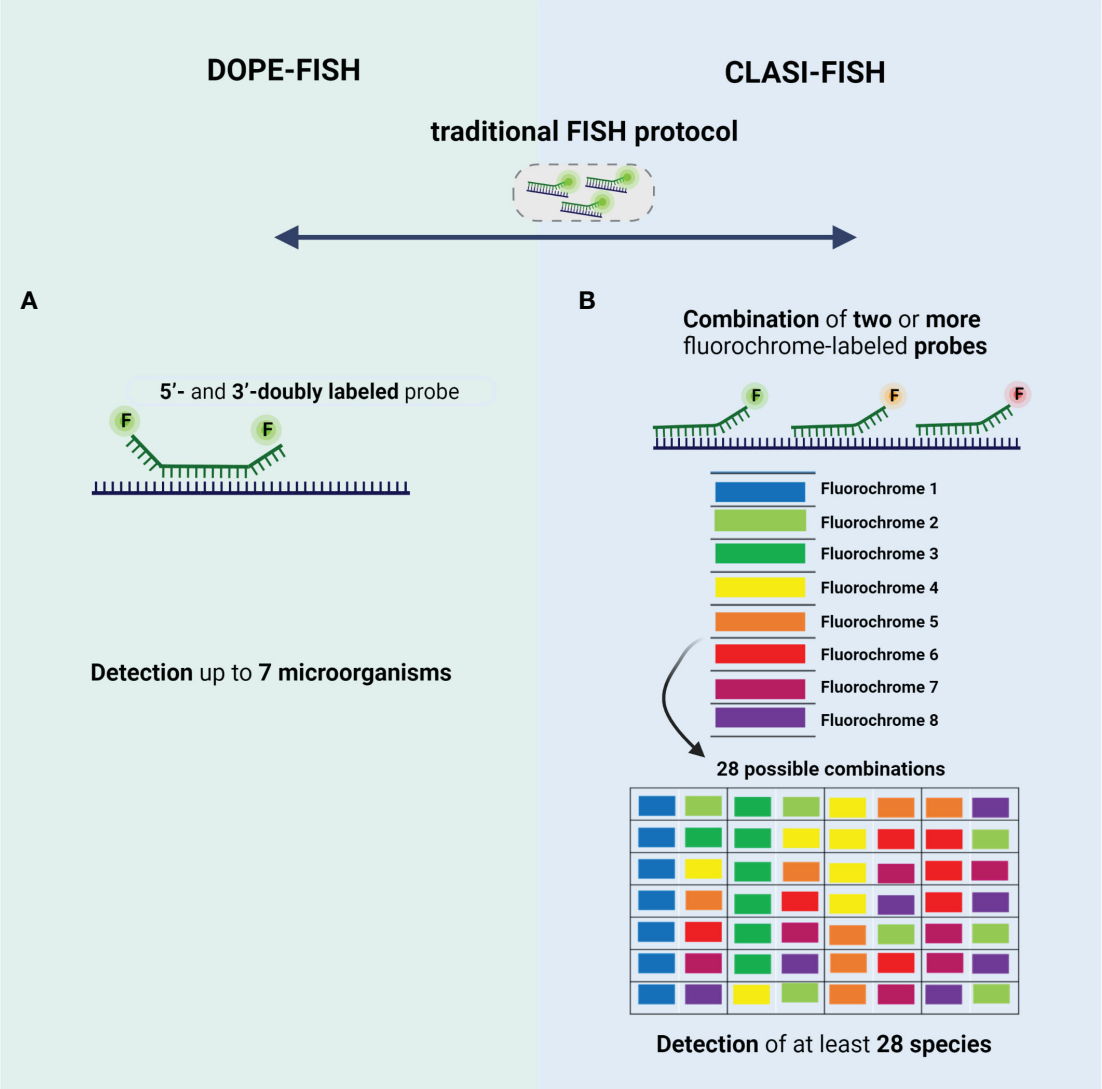
Schematic representation of type of probes used in **(A)** DOPE-FISH and **(B)** CLASI-FISH. The 4 main basic steps of traditional FISH protocol are also common to these FISH variants. **(A)** DOPE-FISH approach uses oligonucleotide probes that are double-labeled with different fluorophores at their 5′- and 3′-end, allowing a detection up to 7 microorganisms; **(B)** CLASI-FISH, microorganisms are hybridized with two or more fluorochrome conjugated probe. Using 8 fluorochromes in this combinatorial labeling approach, it is possible to create up to 28 specific combinations. Created with BioRender.com.

The very limited multiplexing options of FISH were firstly addressed by the development of DOPE-FISH. DOPE-FISH is a straightforward FISH variant which allows to detect up to six microorganisms (Stoecker et al., 2010; Behnam et al., 2012), when a set of 5’- and 3’-doubly labeled probes (probes labeled with two different fluorochromes) is used (**Figure 3A**). In addition, using dual-labeled probes is particulary relevant for environmental microorganisms since the fluorescence intensity signal is almost twice that of traditional FISH, without affecting the specificity (Stoecker et al., 2010; Escudero et al., 2018). The application of DOPE-FISH was firstly reported by Behnam et al., for the *in situ* visualization of a biofilm, involving six different microorganisms, developed on marine sponge. These authors have found that members of *Poribacteria* and *Chloroflexi* phylum, *Nitrospira* genus, *Deltaproteobacteria* and *Gammaproteobacteria* class, and *Archaea* domain, were perfectly detected and located in a unique FISH experiment, showing that *Archaea*, *Poribacteria*, and *Gammaproteobacteria* are the most prevalent populations (Behnam et al., 2012). Later, Heim et al., evaluated the composition of living *Frutexites*-like biofilms using DOPE-FISH and observed that bacteria were concentrated in distinct areas of the biofilm, whereas the Archaea were evenly abundant in the upper and deeper layers of the biofilm (Heim et al., 2017). In 2018, Escudero et al., also applied the DOPE-FISH in samples from a subsurface hard rock samples to analyze the prokaryotic diversity and spatial distribution without compromising the integrity of biofilms. However, in this study, a EUB338 I-III probe (specific for bacteria domain) and a ARC915 probe (specific for Archaea domain) were used instead of a mix of species-specific probes, which means that the species present in biofilm was not identified. In fact, it was only possible to observe the presence of a mixture of microorganisms from bacteria and archaea domains participating in these biofilms (Escudero et al., 2018).

DOPE-FISH, however, cannot reach a higher number of microorganisms simultaneously due the high overlapping excitation and emission spectra (Zimmermann et al., 2014; Lukumbuzya et al., 2019). This is an importante limitation when the characterization of more complex multispecies biofilms found in nature (e.g., biofilms in the oral cavity, airways of patients with cystic fibrosis, intestinal and urinary tract), is intended. In part this might explain the limited number of studies that have applied the DOPE-FISH.

#### 2.2.2 CLASI-FISH

To increase the number of microorganisms that can be detected simultaneously, the CLASI-FISH emerged (**Figure 3B**). This technique developed by Valm et al., allows the distinction of a high number (tens to potentially hundreds) of different microorganisms in a single FISH experiment. In fact, Valm et al. demonstrated that it is possible to detect up to 120 different targets in a unique FISH experiment (Valm et al., 2016). In this approach, the species might be labelled with a combination of two (or more) versions of the sequence probe, each version conjugated to a different fluorochrome (Valm et al., 2012). However, the probes compete by the same target site, which leads a loss of fluorescence intensity signal; hence, cells with low ribosome content might not not be correctly identified (Behnam et al., 2012). This problem could be solved if two (or more) different probes labeled to a different fluorochrome, targeting different regions of rRNA, are used (Valm et al., 2012). However, in this approach the number of probes is higher, and thus a hard probe design and selection might be done to ensure a set of probes that work in well-controlled experiments (e.g., same hybridization temperature). For the image acquisition, the spectral imaging (SI) is used to separate highly overlapping excitation and emission spectra’ fluorochromes (Valm et al., 2011; Valm et al., 2012); for that, a CLSM equipped with multi-anode spectral detectors is used to record the full spectrum at every pixel of the fluorescence image (Garini et al., 2006). Then, a linear unmixing algorithm is applied on spectrally recorded image data to determine the relative contribution from each fluorochrome for every pixel of the image using appropriate reference spectra library of fluorochromes (Valm et al., 2016; Azevedo et al., 2022). The first application of CLASI-FISH was reported by Valm et al, who visualize the spatial distribution of 15 microorganisms in a natural biofilm from the human dental plaque (Valm et al., 2011). These authors, using genus- and family-specific probes, have proved the utility of CLASI-FISH on the study of spatial distribution of a biofilm from a semidispersede human dental plaque. This biofilm was dominated by early colonizers, including species of *Streptococcus*, *Prevotella*, *Actinomyces*, and *Veillonella*. In addition, species belonging to *Prevotella* and *Actinomyces* genera showed the most relevant species for the establishing and maintaining of biofilm complexity (Valm et al., 2011). Then, CLASI-FISH was applied in intact supragingival plaque for a more detailed *in situ* analysis of spatial arrangements of biofilms in three dimensions (Mark Welch et al., 2016). Welch et al., have observed the presence of structures, termed hedgehogs, constituted by *Corynebacterium* filaments and *Streptococcus* at the periphery and *Porphyromonas*, *Haemophilus*/*Aggregatibacter*, *Neisseriaceae*, *Fusobacterium*, *Leptotrichia*, *Capnocytophaga*, and *Actinomyces* within hedgehog structures (**Figure 1**-II) (Mark Welch et al., 2016). These authors also visualize a cauliflower structure in plaque with *Lautropia* at the center and enclosed by *Streptococcus*, *Haemophilus*/*Aggregatibacter*, and *Veilonella* species (Mark Welch et al., 2016). Recently, CLASI-FISH was applied by Schlundt et al., for the quantification and observation of spatial relationships of 7 species in biofilms formed on the surface of plastic marine debris (Schlundt et al., 2020). Concerning the spatial organization, these authors have found some clusters of *Rhodobacteraceae* and filamentous of *Bacteroidetes* with bacteria and phytoplankton distributed irregularly on the plastic surfaces. On the other hand, the quantification of biofilm community showed that *Rhodobacteraceae* and *Bacteroidetes* dominates the surface after during the first week, and afterwards an increase of *Alphaproteobacteria* (other than *Rhodobacteraceae*) and *Gammaproteobacteria* was observed (Schlundt et al., 2020). In all these studies, CLASI-FISH seems to be a straightforward approach for multitargeting; however, it requires the design of multiple probes to work in an equal efficiency under the same hybridization conditions. Recently, Azevedo et al, have demonstrated that using different DNA probes, the thermodynamic parameters (e.g., melting temperatures) are very diverse (Azevedo et al., 2022). As such, these authors have combined NAM-FISH with spectral imaging (SI) for the detection of multiple clinical pathogens simultaneously. In fact, the use of NAMs greatly simplifies the probe design (intercalation of LNA nucleotides with 2’OMe or other NAMs), resulting in a thorough control of the thermodynamics parameters such as melting temperature and Gibbs free energy change (Azevedo et al., 2015; Teixeira et al., 2021; Azevedo et al., 2022). Despite the lack of studies applying the SI-NAM-FISH to biofilm characterization, the robustness and accuracy of this technique is promising.

### 2.3 FISH-based techniques for the study of metabolic activity of biofilm cells

As mentioned in previous sections, several FISH variants provide important phylogenetic information in complex and heterogeneous biofilms (Swidsinski et al., 2005). However, biofilms exhibit dynamic processes that change according with a different stimulus (Römling and Balsalobre, 2012; Franklin et al., 2015; Flemming et al., 2016); in addition, the phylogenetic identification does not provide any metabolic details of the identified microorganisms (Azeredo et al., 2017; Costa et al., 2017). In this sense, the FISH evolution has provided a possibility to identify, locate, and characterize complex communities to the functional level, with the development of some methods such as MicroAutoRadiography (MAR-FISH) (Lee et al., 1999), Nanoscale secondary ion mass spectrometry (FISH-NanoSIMS) (Li et al., 2008) and Bio-orthogonal noncanonical amino acid tagging (BONCAT-FISH) (Hatzenpichler et al., 2014).

#### 2.3.1 MAR-FISH

**Figure 4:**
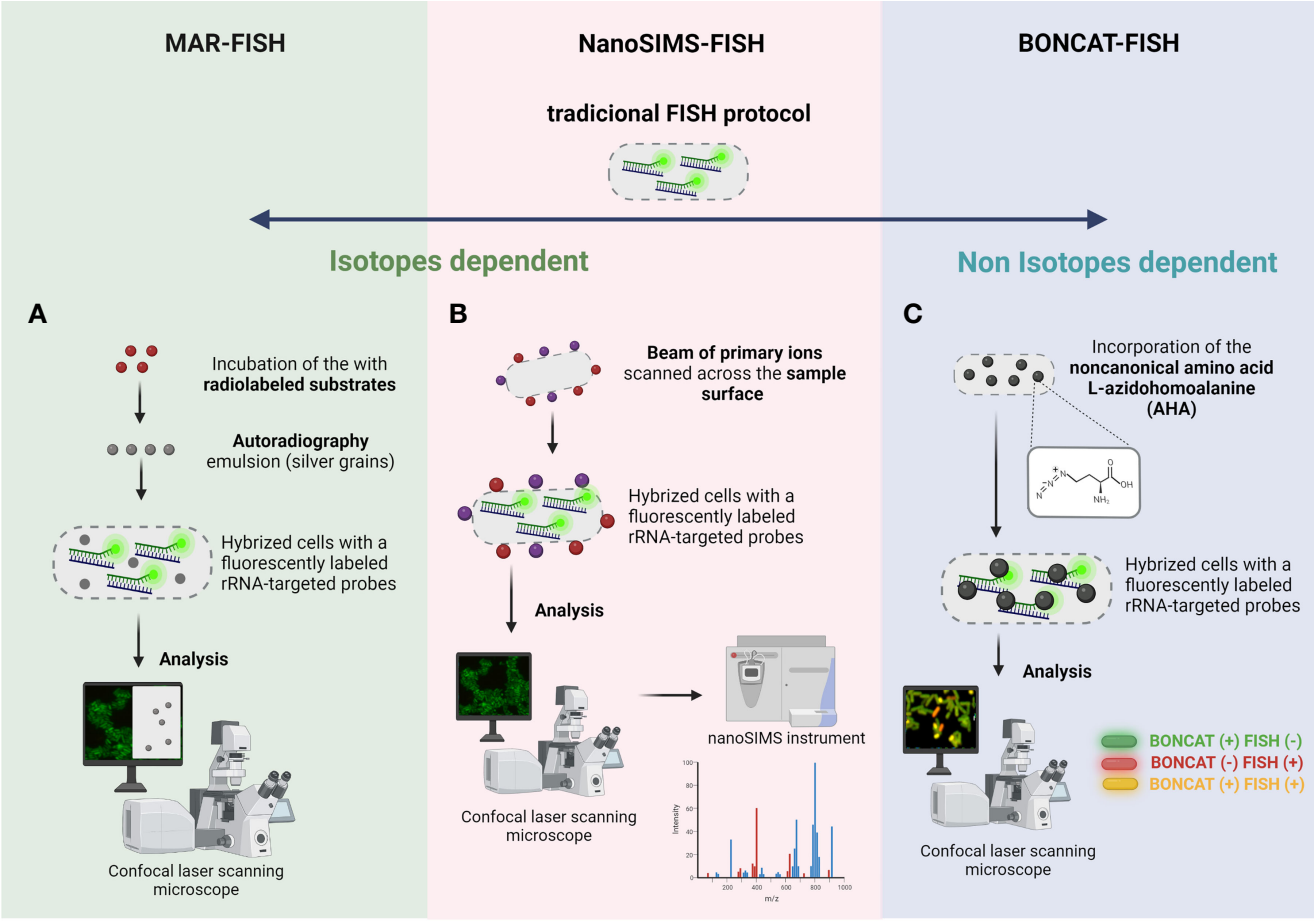
Schematic representation of **(A)** MAR-FISH, **(B)** NanoSims-FISH and **(C)** BONCAT-FISH. The 4 main basic steps of traditional FISH protocol are also common to these FISH variants. **(A)** MAR-FISH and NanoSims-FISH are based on the incorporation of radiolabelled compounds and hybridization of a fluorescently labeled rRNA-targeted probe to examine the phylogenetic identity and the metabolic activity of cells. In MAR-FISH, the FISH and MAR signal is visualized simultaneously by a CLSM. For NanoSIMS-FISH, the hybridized cells are analyzed by CLSM and subsequently the analysis of metabolic activity is obtained in a NanoSIMS instrument. **(C)** In BONCAT-FISH approach, the BONCAT assay (*in vivo* incorporation of the non-canonical amino acid L-azidohomoalanine; green cells in figure) is combined with a fluorescently labeled rRNA-targeted probes (FISH assay; red cells in figure), and the cells are analyzed/visualized by CLSM. Created with BioRender.com.

MAR is a powerful tool, that allows the determination of the uptake of specific radioisotopes by individual microorganisms (Hesselsoe et al., 2005) and has been used to study the *in situ* metabolic activity of microorganisms (Lee et al., 1999; Nielsen et al., 2002a). This method is based on the assimilation of a radiolabeled substrate by individual cells, visualized by exposure to a radiation-sensitive silver halide emulsion placed on top of the radiolabeled bacteria and then processed by standard photographic techniques (Nielsen and Nielsen, 2010). The presence of silver grains reveals that these cells could incorporate the radioactively labeled substrate into the biomass under the chosen incubation conditions (Nielsen, 2021). This technique when combined with FISH, an approach named MAR-FISH (**Figure 4A**), allows to visualize and identify microorganisms (using fluorescently labeled rRNA-targeted probes) and analyze their metabolic activity simultaneously with a CLSM using the fluorescence (for FISH signal) and transmitted light mode (for MAR signal) (Okabe et al., 2004; Nielsen, 2021). This approach has already been used to study some mechanisms in multispecies biofilms in wastewater treatment systems (Okabe et al., 2011). For example, Kindaichi et al., showed that an autotrophic nitrifying biofilm community, was an efficient food web (carbon metabolism), which ensured maximum utilization of soluble microbial products produced by nitrifiers and prevented the buildup of metabolites or waste materials of nitrifiers to significant levels (Kindaichi et al., 2004). Another study performed by Nielsen et al., 2002b was based on detection of the abundance of iron reducers in activated sludge, where around 20% were identified as gamma *Proteobacteria*, and 10% were assigned to the delta *Proteobacteria* (Nielsen et al., 2002b). However, MAR-FISH presents some limitations related with the impossibility to detect several labelling isotopes simultaneously, low single-cell resolution in dense microbial aggregates, the risk of radiation exposure, the limitation to radioisotopes with a suitable half-life and impossibility to monitor the nitrogen or oxygen (Okabe et al., 2004; Behrens et al., 2008).

#### 2.3.2 FISH-NanoSIMS

The limitation of MAR-FISH regarding the types of radioactive elements and the low accuracy of quantification is overcome with NanoSIMS-FISH approach (Gao et al., 2016). NanoSIMS is a dynamic SIMS method in which a beam of primary ions scanned across the sample surface produces secondary ions that reveal the isotopic and elemental composition of the sample (Nunez et al., 2017). This technology allows the determination of ion distributions and the dynamics of different processes, using trace element analysis both of natural and isotopically enriched elements, including carbon, nitrogen, oxygen, magnesium, silicon, sulfur, and iodine in single cells, with a high resolution (e.g., 50 nm) and sensitivity (Slaveykova et al., 2009). NanoSIMS has proven to be an essential tool in microbiology, since it offers information about the nutrient uptake and metabolism of individual microorganism. NanoSIMS can be combined with FISH, in one technique called FISH-NanoSIMS (**Figure 4B**), to directly link the cell identity and location to their activity (Fike et al., 2008; Musat et al., 2012). However, this analysis is not performed simultaneously; first, an epifluorescence microscopy or an CLSM is used to identify and locate the microorganisms directly on samples. Then, the regions of interested will be analyzed by nanoSIMS instrument (Behrens et al., 2008; Kitzinger et al., 2021). Moreau et al., performed a study focused on sulfate-reducing bacteria collected from biofilms, showing that aggregation induced by extracellular metal-binding polypeptides and proteins play an important role in limiting nanoparticle dispersal in natural environments (Moreau et al., 2007). Another interesting application of FISH-NanoSIMS was performed by Stuart et al., where they showed that biofilm cyanobacteria are successful competitors for carbon and nitrogen and that cyanobacterial nutrient and energy requirements control the use of extracellular organic matter (Stuart et al., 2016). FISH-NanoSIMS provides some advantages including imaging at sub-micron resolution while maintaining high mass resolution, isotope analysis; however, this technology implies a complex and expensive sample preparation (embedded process and coating, which roughly takes several days) and a costly equipment (Gao et al., 2016).

#### 2.3.3 BONCAT-FISH

All the techniques mentioned until now are dependent on isotopes and an alternative approach for studying microbial ecophysiology is the BONCAT-FISH that relies on the of chemically modifiable analogs of biomolecules. This technique is based on the *in vivo* incorporation of the noncanonical amino acid L-azidohomoalanine (AHA), which is a surrogate for L-methionine, followed by fluorescent labeling of AHA-containing cellular proteins by azide-alkyne click chemistry (Hatzenpichler et al., 2014). This technique when combined with FISH (**Figure 4C**) enables a link between *in situ* identification and translational activity in environmental biofilms at a single-cell level (Hatzenpichler et al., 2014; Hatzenpichler et al., 2016). Hatzenpichler et al. applied this technology in an environmental context, and probe translational activity of microbial consortia catalyzing the anaerobic oxidation of methane (AOM), a dominant sink of methane in the ocean (Hatzenpichler et al., 2016). In fact, this approach offers the possibility to study microbial activity *in situ* at the individual cell level in a selective, sensitive, and rapid way; however it is an expensive and time-consuming technique (Hatzenpichler et al., 2014).

## 3 Future challenges of FISH in spatially locating biofilm cells and genes

As already mentioned throughout this review, microbial biofilms frequently exhibit rich taxonomic diversity and different spatial organization, which enables diverse metabolic states and social interactions (Nadell et al., 2016; Stacy et al., 2016). In fact, FISH approaches to link the cell identity and location to its function is essential. Specifically, Schaible et al., in a recent work, have combined stable isotope probing (SIP), FISH, scanning electron microscopy (SEM, for, confocal Raman microspectroscopy (Raman), and NanoSIMS in a novel technique called SIP-FISH-Raman-SEM-NanoSIMS, for an *in situ* characterization of an artificial biofilm composed of *E. coli* and *Methanosarcina acetivorans* in terms of taxonomic identity, structure, physiology, and metabolic activity (Schaible et al., 2022). Despite the limited use of SIP-FISH-Raman-SEM-NanoSIMS in biofilm communities, it could be applicable to most biofilm samples (Schaible et al., 2022).

On the other hand, information on spatial genomics has not been possible so far. Single-molecule fluorescence *in situ* hybridization (smFISH) based technologies have been developed to quantitatively measure RNA expression and to assess rRNA spatial localization by directly imaging individual RNA molecules in single cells (Raj et al., 2008; So et al., 2011). Although this approach has high sensitivity and subcellular spatial resolution, is limited to measuring the expression of only a few genes at a time (So et al., 2011). To overcome this limitation a sequential hybridizations (seqFISH) multiplex was developed by Lubeck et al., that allowed to analyze hundreds and even thousands of genes within the same sample at a sub-micron resolution (Lubeck et al., 2014). The individual transcripts in cells are barcoded by sequential rounds of hybridization, imaging, and probe stripping. As the transcripts are fixed in cells, the corresponding fluorescent spots remain in place during multiple rounds of hybridization and can be aligned to read out a fluorochrome sequence (Lubeck et al., 2014). However, seqFISH is time consuming, and Multiplexed Error-Robust FISH (MERFISH) was published in 2015, as a method for reliably measure the expression and spatial position of thousands of different transcripts concurrently (Chen et al., 2015). A specific coding probe consisting of a complementary sequence and two flanking readout sequences is used. This approach detects the analyte through several rounds of hybridization within 15 min compared with contemporary methods which required over 10 h for the analysis of mRNA molecules (Chen et al., 2015). Regarding to 16S rRNA labeling, Shi et al., introduced a High-Phylogenetic-Resolution microbiome mapping by FISH (HiPR-FISH) (Shi et al., 2020). This high multiplexity technology use binary encoding, spectral imaging and decoding based on machine learning to create micrometre-scale maps of the locations and identities of hundreds of microbial species in complex communities. This approach is based on two steps of hybridization: the first step uses taxon-specific probes modified with DNA flanking sequences, and the second step uses fluorescently labeled readout probes that target the flanking sequences (Shi et al., 2020).

Recently, Dar et al., performed the first study with this modern transcriptome-imaging technique to understand the microscale organization of microbial populations and communities (Dar et al., 2021). In this study, a novel multiplex approach, namely parallel seqFISH (par-seqFISH), was introduced for the study of a range of *Pseudomonas aeruginosa* physiological conditions as planktonic and biofilm cells. The par-seqFISH allowed a simultaneous and efficient 16S rRNA identification and measurement of mRNA expression of hundreds of genes within individual cells. Hence, the metabolic and cellular states related to bacterial virulence, as well as the phenotype in different stages of growth was explored, highlighting the importance of understanding the roles that spatial and temporal heterogeneity plays in microbial populations.

Overall, the FISH variants described in this review showed that *in situ* visualization of multispecies biofilms (in terms of position/location of biofilm cells and genes) have received increasing attention in different fields, ranging from healthcare, environment and industry. The improvement of the accuracy of FISH technologies such as CLASI-FISH and HiPR-FISH (for a multiplexed spatial detection of microorganisms), BONCAT-FISH (for the study of microbial activity *in situ*), SIP-FISH-Raman-SEM-NanoSIMS (for the *in situ* identification and simultaneously analysis of structure, physiology, and metabolic activity of cells), and par-seqFISH (for the spatial expression of various genes), enables us to understand the interaction among microorganisms, metabolites, genes in biofilms communities from an ecological point of view, by looking directly inside biofilm architecture. FISH-based techniques couple with other cutting-edge technologies such as artificial intelligence is very promising. In fact, recent advances on machine learning (ML) (a branch of artificial intelligence), in terms of resolution and image analysis improvements, already showed promising results in the diagnostic of some biofilm-related infections (Dimauro et al., 2020; Xu et al., 2020). In fact, ML can be used to analyze biofilms in a variety of ways. For instance, ML algorithms might be used to analyze data from gene expression studies of biofilms; analyzing patterns in gene expression, new strategies to target biofilm infections can be explored (Rajput et al., 2022). On the other hand, ML might also be used to model the growth and behavior of biofilms, allowing researchers to predict how biofilms will respond to different conditions. For example, ML was already used to predict how biofilms respond to a specific antimicrobial agent (Artini et al., 2022). Another potential approach is to use ML to analyze images of biofilms obtained through FISH technique and CLSM (Gu et al., 2022); using ML algorithms to analyze FISH images, researchers can potentially identify patterns and correlations that would be difficult or impossible to detect using traditional methods. For example, ML algorithms could be optimized to analyze FISH images and identify specific microbial species or groups based on their fluorescence patterns (e.g., areas where specific microorganisms are present). The algorithm could then be used to automatically analyze new images and identify regions that match the annotations. This can further allow researchers to quantify the abundance and distribution of different microorganisms within a biofilm and gain insights into their interactions and dynamics. ML could also be used to analyze FISH images in real-time, allowing researchers to monitor changes in microbial abundance and distribution over time. This can be particularly useful for analyzing the effects of different stimulus (e.g., temperature, pH, nutrients, antimicrobial agents) on biofilm growth and structure in real-time. Overall, ML has the potential to be a powerful tool for analyzing biofilms and gaining new insights into their structure, function, and behavior. In the near future, it will be exciting to see the application of these advanced technologies for the spatial and temporal characterization of biofilms, in particular the real-time monitoring of the species and genes expression during the biofilm formation, development or eradication.

## Author contributions

AB, SM, LC and ASA contributed to the conception and design of the study. AB, SM and ASA wrote the manuscript. ASA and AB prepared the figures. ASA, LC and NFA have read and agreed to the published version of the manuscript.
